# Adverse Drug Events in Patients with Dementia and Neuropsychiatric/Behavioral, and Psychological Symptoms, a One-Year Prospective Study

**DOI:** 10.3390/ijerph16060934

**Published:** 2019-03-15

**Authors:** Marta H. Hernández, Conxita Mestres, Pilar Modamio, Jaume Junyent, Lluís Costa-Tutusaus, Cecilia F. Lastra, Eduardo L. Mariño

**Affiliations:** 1 Pharmacy Department, Grup Mutuam, 08024 Barcelona, Spain; martahh1@blanquerna.url.edu; 2 School of Health Sciences Blanquerna, University Ramon Llull, 08025 Barcelona, Spain; lluisct@blanquerna.url.edu; 3 Clinical Pharmacy and Pharmacotherapy Unit, Department of Pharmacy and Pharmaceutical Technology, and Physical Chemistry, Faculty of Pharmacy and Food Sciences, University of Barcelona, 08028 Barcelona, Spain; pmodamio@ub.edu (P.M.); ceciliafernandez@ub.edu (C.F.L.); emarino@ub.edu (E.L.M.); 4 Mutuam Güell Hospital, 08024 Barcelona, Spain; jaume.junyent@mutuam.com

**Keywords:** psychogeriatrics, drug safety, neuropsychiatric/behavioral and psychological symptoms, falls, dementia

## Abstract

Older people usually present with adverse drug events (ADEs) with nonspecific symptoms such as cognitive decline, recurrent falls, reduced mobility, and/or major deterioration. The aims of this study were to assess the ADEs of patients with dementia and presenting neuropsychiatric/behavioral, and psychological symptoms in dementia (BPSD) and to categorize and identify the principal factors that allow to prevent ADEs, and separately ADEs that result in falls. To that end, a one-year prospective study in a psychogeriatric ward (July 2015 to July 2016) was performed. All patients admitted to this ward were eligible for enrolment. Patients who met any of the following criteria were excluded from the study: Patients without cognitive impairment, a length of stay under 7 days, and palliative or previous psychiatric pathology. We included 65 patients (60% women, 84.9 years ± 6.7) with mild to moderate cognitive impairment, moderate to severe functional dependence, and a high prevalence of geriatric syndromes and comorbidity. A total of 87.7% were taking five or more drugs (mean 9.0 ± 3.1). ADEs were identified during the interdisciplinary meeting and the follow up by clinical record. Sixty-eight ADEs (81.5% patients) were identified, of which 73.5% were not related to falls. From these, 80% were related to drugs of the nervous system. The Naranjo algorithm determined that 90% of ADEs were probable. The severity of the ADEs was Category E in 34 patients (68%). The number of preventable ADE according to the Schumork–Thornton test was 58%. The main ADE was drowsiness/somnolence (27.7%). ADEs related to falls represented a 26.5%. The balance between effective treatment and safety is complex in these patients. A medication review in interdisciplinary teams is an essential component to optimize safety prevention.

## 1. Introduction

One of the issues that most concerns patients, caregivers, health professionals, and the health system is drug safety. The epidemiology of adverse drug events (ADEs) suffered by patients has been described by various studies [1,2,3]. ADEs are a common cause of hospitalization and are more frequent in the elderly [4]. Although there is less information during hospitalization, a meta-analysis and a systematic review reported that around 11% of all hospitalized adults experience an ADE, with 2.1% reported as serious [5,6].

The diagnosis of ADEs in geriatric patients can be complex because older people usually present with high levels of multimorbidity and polypharmacy. Furthermore, ADEs often occur with nonspecific symptoms such as cognitive decline, recurrent falls, reduced mobility, and/or major deterioration. Geriatric syndromes being a challenge that cannot be detected as caused by an ADE, it can be challenging to discern if medications have been involved or not. 

ADEs have major clinical and economic consequences: They prolong hospital stay [7,8,9,10], increase the use of resources, and they can lead to fatal consequences, ranging between the fourth and sixth leading cause of death [5,6]. It has been suggested that approximately between 27.6% and 51% [11] of ADEs could be preventable [11,12,13]. 

Few interventions have proven to be effective; however, identifying and reducing inappropriate prescribing has the potential to decrease the incidence of ADEs [14]. 

An important ADE in elderly patients is falls, which are the dominant cause of injury-related emergency room visits and injury-related deaths among older adults [15]. Around a third of older adults experience at least one fall annually, and 1 out of 55 of these falls causes injury [16]. Furthermore, fall injuries are the 20th most expensive medical conditions among community-dwelling older adults and are associated with premature institutionalization, a decreased quality of life, impaired mobilization, and a substantial rise in health care costs [17,18]. 

The use of certain medications is recognized as a major risk factor for falls. Psychotropic medication has been consistently reported to increase this risk [19,20,21,22,23]. A literature review and meta-analysis [24] indicated consistent associations between the use of antipsychotics, antidepressants, and benzodiazepines with an increased risk of falls in older adults. Additionally, some studies have shown an association between the use of antihypertensive medication, digoxin, type I antiarrhythmics, and diuretics with falls [25,26].

No studies that allow continuous monitoring and evaluation to determine the safety of the use of medicines in a special geriatric group and in a context of real practice have been found. Thus, the aims of the present study were to assess the ADEs of patients with dementia and presenting neuropsychiatric/behavioral and psychological symptoms in dementia (BPSD) and to categorize and identify the principal factors that allow to prevent ADEs, and separately ADEs that result in falls.

## 2. Methods

### 2.1. Design and Study Setting

This was a one-year observational and prospective study conducted in a psychogeriatric ward (21 beds) in an intermediate care hospital: HSS Mutuam Güell (165 beds) in Barcelona, Spain. 

The study was carried out from July 2015 to July 2016. All patients admitted to this ward were eligible for enrolment. Patients who met any of the following criteria were excluded from the study: Patients without cognitive impairment, a length of stay under 7 days, and palliative or previous psychiatric pathology. The present study was designed following the recommendations of the STROBE statement guidelines for reporting observational studies [27].

### 2.2. Definitions

To assess the functional and cognitive impairment, we used different indices and tests:

- The Barthel index, a scale that measures disability or dependence on activities of daily living. Its values range between 0 and 100, with the lowest score indicating a high dependency [28].
- The global deterioration scale (GDS) determines cognitive function. It has 7 different stages: Stages 1–3 are the pre-dementia stages, and stages 4–7 are the dementia stages [29].

An ADE was defined as any injury, mild or severe, caused by the therapeutic use (including non-use) of a medication [30]. The Naranjo algorithm was used for the causality assessment of ADEs. The ADE probability scale consists of 10 questions that are answered as either Yes, No, or “Do not know”. Different point values (−1, 0, +1 or +2) are assigned to each answer. Total scores range from −4 to +13; the reaction is considered definite if the score is 9 or higher, probable if 5 to 8, possible if 1 to 4, and doubtful if 0 or less [31].

The Schumock–Thornton test was performed for the preventability assessment of suspected ADEs. Twelve questions compose the test and if the answer is yes in one or more of these questions, the ADE can be considered preventable [32]. 

In relation to falls, the three tools used were:

- The Tinetti balance assessment: Scoring of the Tinetti assessment tool is done on a three-point ordinal scale with a range of 0 to 2. A score of 0 represents the most impairment, while a score of 2 represents independence. The individual scores are then combined to form three measures: An overall gait assessment score, an overall balance assessment score, and a combined gait and balance score. The maximum score for the gait component is 12 points. The maximum score for the balance component is 16 points. The maximum total score is 28 points. In general, residents who score below 19 are at a high risk of falls. Residents who score in the range of 19–24 points have a risk of falls [33].
- The Downton fall risk index: The Downton fall risk index includes well-documented risk factors for falls and therefore offers satisfactory content validity and also seems to be very easy to administer. The Downton fall risk index includes 11 risk items which are scored at one point each. Scores are summed to give a total index score with a range of 0–11. A score of 3 or more is taken to indicate a high risk of falls [34].
- The anticholinergic burden was determined using the drug burden index (DBI) [35,36]: Anticholinergic burden is defined as the cumulative effect of taking one or more drugs that are capable of producing adverse anticholinergic effects. High scores have been associated with an increased risk of suffering adverse events (including falls, delirium and cognitive disorders). The DBI scale measurement of the anticholinergic effect is based on the calculation of a mathematical formula that takes into account the prescribed dose and the minimum effective dose of the drug.

### 2.3. Ethics

Clinical Research Ethics Committee evaluation: Comitè Ètic d’Investigació Clínica de la Fundació Unio Catalana Hospitals (Ethical Committee for Clinical Research at the Unio Catalana Hospitals Foundation) (CEIC 14/42). In addition, the study was approved by the Spanish Agency of Medicines and Medical Devices with the codification MHH-ANT-2014-01.

### 2.4. Data Collection

ADEs were identified during a weekly meeting that was held between the pharmacist and the physician to review the patient treatment. The follow up of the patient during they stay was done using the clinical record of the patient (Aegerus^®^). Information on other variables was obtained from sources such as electronic prescriptions, electronic medical records (Aegerus^®^), and the Catalonian Health Care System electronic record (HC3). Patients were included in the study in a consecutive way upon admission if they did not meet any of the exclusion criteria (patients without cognitive impairment, a length of stay under 7 days, and palliative or previous psychiatric pathology). In order to continue the study, all the patients/or caregiver were required to sign the consent. 

All data were recorded in a database using Microsoft Excel^®^ (Microsoft, Albuquerque, NM, USA) 2010 and Power Pivot^®^ (Microsoft, Albuquerque, NM, USA). 

The variables included were:

(i) Demographic: Age, gender, and length of stay.
(ii) Pharmacological: Number and type of drugs, dosage, frequency, route of administration, and starting prescription date (if possible). Qualitative classification of drugs under the anatomical therapeutic chemical code (ATC) classification system.
(iii) Clinical: Diagnosis (ICD-10 International Classification of Diseases, 10th Revision), dementia type, geriatric syndromes (falls, Tinetti, and Downton), dysphagia, pain, ulcers, constipation, dyspnea, hearing loss, visual impairment, malnutrition, insomnia, depressive-anxiety syndrome, incontinence, and cognitive assessment (according to the GDS-FAST scale, functional assessment (according to the Barthel Index)).

To assess the ADE, we used the Naranjo algorithm to determine causality. Regarding severity, we used the classification system of the Institute for Healthcare Improvement [37] (and its Spanish version [30]). The Schumock–Thornton algorithm was performed to evaluate preventability. Specifically for the falls group, we used the Tinetti and Downton tests. 

### 2.5. Statistical Analysis

SPSS software (IBM-SPSS 25.0 version, IBM, Armonk, NY, USA) was used for statistical analysis. For the comparison of the quantitative variables, the Kolmogorov–Smirnov test was used to assess the normal distribution of the sample. A student’s t test was used for comparison. 

## 3. Results 

### 3.1. General Characteristics 

Between July 2015 and July 2016, 114 patients were admitted to the unit, of whom 65 patients with dementia and neuropsychiatric/behavioral and psychological symptoms in dementia (BPSD) disturbances were followed. We excluded 49 because even though they were admitted to this unit, they did not meet any criteria of possible dementia or BPSD.

Baseline characteristics (Table 1) showed a mild to moderate cognitive impairment (mean 4.5 ± 1.8), a moderate to severe functional dependence (mean 43.8 ± 23.9), and a high prevalence of geriatric syndromes: Incontinence (*n* = 44, 67.7%), constipation (*n* = 44, 67.7%), previous falls (*n* = 47, 72.3%), and previous fractures (*n* = 12, 18.5%). The most prevalent type of dementia was Alzheimer’s (30.8%), but the cognitive impairment study was not completed for 43.1% of the patients (Table 1).

The most common patient diagnoses included diseases of the circulatory system (83.1%) and endocrine, nutritional, and metabolic diseases (60%) (Table 1). The comorbidity mean was 4.8 ± 1.6; a total of 64.6% of patients had more than four chronic diseases.

A total of 87.7% of the patients were taking five or more drugs (mean of 9.0 ± 3.1 drugs per patient), and 38.5% were taking ten or more. We observed at admission (N-ATC classification) that N05A—antipsychotics were the most frequent (51–78.5% of patients), followed by N05C—hypnotics and sedatives/N05B—anxiolytics (31–47.7%), N06A—antidepressants (35–53.9%), N02—analgesics (43–66.2%), N06D—anti-dementia drugs (24–30.9%), N03A—antiepileptic drugs (8–12.3%) and N04—anti-Parkinson’s drugs (3–4.6%).

Regarding the risk of falls, the Tinetti mean was 15.5 ± 8 and the Downton test mean was 4.5 ± 1.3 (93.8% result of 3 or more); therefore, they had a high risk of falls (Table 1) 

### 3.2. Adverse Drug Events

During the period, we identified 68 ADEs in 53 patients, 81.5% of the total patients included presenting with some form of ADE at admission and/or during the stay. A total of 22.6% of these patients presented with more than 1 ADE (1 patient had 4 ADEs, 1 patient had 3 ADEs, and 10 patients had 2 ADEs). There were no differences when comparing the length of stay between the patients who presented with an ADE (mean length 67.9 days ± 43.1) versus the ones who did not (mean length 71.4 days ± 61.2) (*p* = 0.39). We compared the two groups on the DBI in admission and we found no differences between the group who presented with an ADE (mean 1.1 ± 0.8) and the patients who were ADE-free (mean 1.0 ± 0.7) (*p* = 0.16).

A total of 73.5% of the ADEs were not related to falls. From these, 80% were related to drugs of the ATC nervous system. From this group, 46% (23 ADEs) were attributed to the psycholeptic group (Quetiapine was the most prevalent with 20% (10) of the ADEs related, which included somnolence, weakness, hypoactivity, and akathisia) (Table 2). 

The application of the Naranjo algorithm (except for falls) determined that 1 (2%) of the ADEs was classified as definite, 45 (90%) as probable, and 4 (8%) as possible and doubtful. 

The severity of the ADEs was Category E: 34 (68%) of the cases (temporary harm to the patient who required intervention) and 16 (32%) were category F (temporary harm to the patient who required initial or prolonged hospitalization).

Regarding the preventability and according to the Schumork–Thornton test, 58% (29) of the ADEs (without taking into account ADEs related to falls) were preventable, 6% were possibly preventable (3), and 36% of the ADEs were unavoidable (18). From the preventable ADEs, 50% (16) were reported at admission, in 62% of the cases the ADE was derived when the treatment was stopped, and the rest with a dosage correction. 

The main ADE (Table 3) was drowsiness/somnolence (27.7%), followed by weakness (12.8%) and hypoactivity and hypotension (10.7%), (Table 3). Quetiapine was the drug most frequently related with an ADE.

### 3.3. Adverse Drug Events-Falls 

A particular group of concern are the patients who suffered falls during their stay in the psychogeriatric unit, so we performed a separate analysis of these patients who presented with falls.

There were 18 ADEs related to falls (26.5% of total ADEs) during the stay and affected 26.2% of patients, only 3 (16.7% total falls) of whom were injured, with one patient falling twice.

Regarding the results of the Tinetti test (15.6 ± 8.1 for the 18 patients who had a fall), 10 patients (55.6%) had a score lower than 19, so they had a high risk of falls, 6 patients (33.3%) scored 19–24, and 2 patients (11.1%) scored 24 and therefore had no risk.

According to the Downton Index, the mean value for the 18 patients was 4.5 ± 1.2. A total of 61.1% of patients had no sensorial deficits, and just 11.1% could wander around without difficulties. Fall risk medication classes (Table 4) were highly prevalent, and hypotensors which were not diuretic (72.2%) were the most frequent drugs. A total of 38.89% of the patients were taking four high risk of fall drugs, 33.33% were taking three, 16.67% were taking two, and only one of the patients who fell was not taking any drugs with a fall risk. 

When comparing the tests and indices between patients who fell and those who did not, the Downtown test found no differences between the groups (fall group mean score 4.8 ± 1.0 and the nonfall group 4.4 ± 1.3 *p* = 0.08). When comparing the Tinetti score (fall group mean score 17.4 and nonfall group 14.7 ± 8.7 *p* = 0.12), there was no significant difference in the length of stay (fall group 81.1 ± 62.8 days and nonfall group 65.3 ± 48.3 days, *p* = 0.14), although the difference in means was higher in the fall group (15.7 days). We found no significant differences regarding the anticholinergic burden at admission, measured with the DBI (mean score fall group was 1.3 ± 0.8 versus nonfall group 1.0 ± 0.7, *p* = 0.05). 

## 4. Discussion

The primary objective of this study was to analyze the ADEs in patients admitted to a psychogeriatric ward to control BPSD. Different widely employed scales [31,32] were used for the assessment, and we found a high proportion of the ADEs were preventable or possibly preventable (64%), not differing overly from the usual rate that is considered in the elderly that ranges between 27.6% and 51% [11]. A high proportion of the ADEs were identified at admission (50%) [1,13], so the ADEs were probably responsible for the symptoms worsening. When this medication was removed or changed for a more appropriate one, the symptoms reduced or disappeared [38]. This is a highly relevant result for patient safety and shows that a routine systematic review of the pharmacotherapy can result in a better clinical outcome, quality of life, and economic saves [10,15]. These population characteristics are associated with well-known conditions that increase ADEs, such as multiple chronic conditions (4.8 ± 1.6 comorbidities) and polypharmacy (9.0 ± 3.1 drugs per patient), and an associated increase in healthcare utilization, worsening cognitive impairment, associated geriatric syndromes, and mortality [39]. This added to the characteristics of patients with cognitive impairment and BPSD, which makes the patient-centered care team paramount to balancing safety and efficiency. 

There was a difference of 15.7 days longer stay between the fall group and the nonfall group. Despite not being statistically significant, it is important regarding the clinical outcomes and economic impact for the system, with falls in older adults included in the 20 most expensive medical conditions [15,17]. It is a priority to identify the different factors related to the risk of a fall, especially in patients who present with a high risk of falling (93.8% had a high risk of falls according to Downton) as a baseline characteristic. Based on the risk of fall scale used (Tinetti and Downton), we found no differences, so in this population, these scales are not useful to optimize intervention and stratification of risk. The Downton index was developed for elderly people in continuing care wards and it has been validated externally among stroke patients in geriatric rehabilitation [40], in which a moderately high correlation was found between the predicted risk of falls and observed falls; it has also been used in residential care facilities.

Another important factor is the anticholinergic burden [41], with our results showing the DBI had an impact on falls. These results demonstrate that there is a trend that correlates high DBI with increased fall risk. There is a need to adapt the medication of these patients to lower the anticholinergic burden, as a high anticholinergic burden generates poor clinical outcomes. In these psychogeriatric patients, it is important to choose drugs with a lower anticholinergic effect. It has been noted that the anticholinergic effect of antipsychotics differs between drugs, and this characteristic should be considered over the cost, particularly in patients who need high doses. To choose the most appropriate drug regarding its anticholinergic burden is a very wise strategy, and it will be recommendable in these patients, because the adverse consequences outweigh the cost [42,43].

A total of 83.1% of patients had cardiovascular disease, and 72.2% of the patients had experienced a fall in addition to having an ADE affecting the cardiovascular system, with hypotension being the most frequent (10.6%). Therefore, cardiovascular medication should be carefully chosen [44], and a follow up of the prescription is essential to reduce the risk of falls. It is important to know which cardiovascular medication is most associated with falls. Loop diuretics have a well-known correlation, and between antihypertensive drugs, we found heterogeneous characteristics; therefore, we should prioritize drugs with a better correlation of safety or even a protective effect on the risk of falls [24,38]. 

Our study has some limitations. A specific sample size has not been established in order to provide a predetermined power to the statistical analysis, as ours is a sub-study of a cohort whose main goal was to was to determine how multifaceted pharmaceutical intervention based on a medication review and multidisciplinary follow up could improve the treatments and minimize the risk of patients in the psychogeriatric unit [38]. However, the study period was long, and this may mitigate this weakness. 

## 5. Conclusions

In patients with BPSD and the basic risks that a geriatric patient already has, such as polypharmacy and multimorbidity, we must add the high use of psychotropic drugs and cognitive impairment. All of this increases the risk of ADEs in general and particularly the risk of falls, with fatal consequences for the patient and the health system in general. 

Falls as adverse events should be included in the clinical trials of new medication, in order to have reliable evidence of the risk of falls.

In these patients, it is necessary to choose the medication with the lowest anticholinergic load, since there is a direct relationship with an increase in falls, as well as worse cognitive deterioration and a greater presence of BPSD.

A regular systematic review of the pharmacotherapy by an interdisciplinary team can prevent and detect ADEs.

## Figures and Tables

**Table 1:** Baseline characteristics of the patients included.

Variable	Results
Number of patients	65
Age	84.85 years (SD = 6.68; rank = 68–96)
Gender	Women = 39 (60%); men = 26 (40%)
Place of origin	Home = 36 (55.4%); acute hospital = 27 (41.5%); intermediate care = 2 (3.1%)
Length of stay (days)	58. 5 (median)
Geriatric syndromes	Previous falls = 47 (72.30%) previous fractures 12 (18.46%)
	Constipation = 44 (67.7%)
	Depression/anxiety = 21 (32.3%)
	Dysphagia = 21 (32.3%)
	Dyspnea = 3 (4.6%)
	Hearing loss = 10 (15.4%)
	Incontinence = 44 (67.7%)
	Insomnia = 22 (33.8%)
	Malnutrition = 6 (9.2%)
	Pain = 15 (23.1%)
	Ulcers = 16 (24.6%)
	Visual impairment = 21 (32.3%)
Type of dementia	Alzheimer’s disease = 20 (30.8%)
	Vascular = 5 (7.7%)
	Mixed = 3 (4.6%)
	Diagnosis not completed = 28 (43.1%)
	Lewy body = 5 (7.7%)
	Others = 4 (6.2%)
Functional abilities (Barthel Index)	Some dependence or independence (BI 80–100) = 6 (9.2%)
	Slight dependence (BI 60–75) = 16 (24.6%)
	Moderate dependence (BI 40–55) = 18 (27.7%)
	Severe dependence (BI 20–35) = 12 (18.5%)
	Total dependence (BI 0–15) = 13 (20%)
Cognitive function (Global deterioration scale (GDS))	Incipient (GDS 3) = 16 (24.6%)
	Mild (4) = 18 (27.7%)
	Moderate (5) = 16 (24.6%)
	Severe (6) = 13 (20.0%)
	Very severe (7) = 2 (3.1%)
Comorbidity	Certain infectious and parasitic diseases 3 (4.6%)
	Diseases of the blood and blood-forming organs and certain disorders involving the immune mechanism 10 (15.4%)
	Diseases of the circulatory system 54 (83.1%)
	Diseases of the digestive system 15 (23.1%)
	Diseases of the ear and mastoid process 5 (7.7%)
	Diseases of the eye and adnexa 11 (16.9%)
	Diseases of the genitourinary system 21 (32.3%)
	Diseases of the musculoskeletal system and connective tissue 19 (29.2%)
	Diseases of the nervous system 18 (27.7%)
	Diseases of the respiratory system 8 (12.3%)
	Diseases of the skin and subcutaneous tissue 1 (1.5%)
	Endocrine, nutritional and metabolic diseases 39 (60%)
	Injury, poisoning and certain other consequences of external causes 17 (26.2%)
	Mental and behavioral disorders 10 (15.4%)
	Neoplasms 11 (16.9%)
	Symptoms, signs and abnormal clinical and laboratory findings, not elsewhere classified 9 (13.8%)

**Table 2:** Distribution of adverse drug effects (ADEs) by the main anatomical group, therapeutic group, and drug.

Anatomical Main Group	Therapeutic Subgroup	Drug	n ADE	%
A—Alimentary tract and metabolism			2	4
	Drugs for acid related disorders		1	2
		Ranitidine	1	2
	Drugs used in diabetes		1	2
		Metformin	1	2
C—Cardiovascular system			8	16
	Agents acting on the renin-angiotensin system		1	2
		Losartan	1	2
	Antihypertensives		2	4
		Doxazosin	2	4
	Calcium channel blockers		2	4
		Diltiazem	1	2
		Nifedipine	1	2
	Cardiac therapy		2	4
		Digoxin	1	2
		Nitroglycerin (patches)	1	2
	Diuretics		1	2
		Furosemide	1	2
N—Nervous system			40	80
	Analgesics		7	14
		Fentanyl	2	4
		Morphine	1	2
		Pizotifen	1	2
		Tramadol	3	6
	Antiepileptics		3	6
		Valproic acid	1	2
		Clonazepam	2	4
	Psychoanaleptics		7	14
		Citalopram	1	2
		Donepezil	3	6
		Galantamine	1	2
		Mirtazapine	1	2
		Venlafaxine	1	2
	Psycholeptics		23	46
		Haloperidol	2	4
		Lormetazepam	1	2
		Lorazepam	2	4
		Midazolam	1	2
		Olanzapine	1	2
		Quetiapine	10	20
		Risperidone	6	12

**Table 3:** Distribution of adverse drug events (ADEs), except falls.

ADE	*n*	%
Drowsiness/somnolence	13	27.7
Weakness and hypoactivity	6	12.8
Hypotension	5	10.6
Syncope	4	8.5
Agitation	3	6.4
Hallucinations	3	6.4
Delirium	3	6.4
Akathisia	2	4.3
Hypoglycemia	2	4.3
Constipation	2	4.3
Nausea and vomiting	1	2.1
Diarrhea	1	2.1
Tremor	1	2.1
Priapism	1	2.1

**Table 4:** Distribution of high-risk medications for falls by the Downton scale.

Drug Class	Number of Patients under Prescription	%
Hypotensors not diuretics	13	72.2
Antidepressants	11	61.1
Antipsychotics	10	55.6
Benzodiazepines	9	50.0
Diuretics	7	38.9
Antiparkinsonians	3	16.7
